# Melanoma: From Research to Treatment

**DOI:** 10.1155/2011/710697

**Published:** 2012-04-10

**Authors:** Paolo A. Ascierto, John M. Kirkwood, Francesco M. Marincola, Giuseppe Palmieri

**Affiliations:** ^1^Unit of Medical Oncology and Innovative Therapy, Department of Melanoma, National Tumor Institute, 80131 Naples, Italy; ^2^Division of Hematology/Oncology, Department of Medicine, University of Pittsburgh Cancer Institute, Pittsburgh, PA 15232, USA; ^3^Department of Transfusion Medicine, NIH Clinical Center, National Institute of Health, Bethesda, MD 20892, USA; ^4^Institute of Biomolecular Chemistry-CNR, Trav. La Crucca, 3-Baldinca Li Punti, 07100 Sassari, Italy

The last two decades have deepened our understanding of the biology of melanoma in significant new directions. This progress has resulted in the development of therapeutics with unprecedented clinical potential. Ipilimumab and tremelimumab are anti-CTLA4 monoclonal antibodies that alter the regulatory immune network responsible for damping anticancer immune responses, and this class of agent has shown the first significant impact upon survival of metastatic melanoma. Meanwhile, agents that inhibit activating mutations that drive the oncogenic processes such as B-RAF or c-KIT have shown clear effectiveness and almost immediate responses. It remains uncertain how the current individual treatments work at the level of the tumor during treatment. Moreover, the weight that the genetic background of the host may have in relation to treatment response has yet to be explored. A lot still needs to be done to improve the durability of treatment benefits, and cure remains an elusive goal. The topics presented here are not an exhaustive representation of the field of melanoma research, but a sampling of the large and many-faceted agenda of current interest that we have the pleasure of sharing with the readers. We would like to thank the authors for their excellent contributions and patience in this venture. Finally, the fundamental work of reviewers of the papers is also very warmly acknowledged.

This special issue contains seven papers, three related to epidemiology, diagnosis, risk factors, and characteristics of melanoma. Two papers focus on specific disease treatments, one using the isolated limb infusion (ILI) and the second based on the adoptive transfer of autologous tumor-infiltrating lymphocytes (TIL) for therapy of locally or systemically advanced melanoma, respectively. Also two papers address the management of brain metastases and uveal melanoma.

In the paper entitled *“The contribution of electron paramagnetic resonance to melanoma research*,” Q. Godechal and B. Gallez showed how electron paramagnetic resonance (EPR), a method able to detect free radicals trapped in melanin pigments, has recently provided insight to basic features of melanoma that may improve its diagnosis. These advances may improve the diagnosis of melanoma, but the limitations of the method are also detailed.

The paper entitled “*Nonsteroidal anti-inflammatory drugs and risk of melanoma*” presents a pilot study of the association between NSAID usage and melanoma incidence, to determine whether epidemiologic evidence of a chemopreventive effect of these agents is compelling. On the basis of the conflicting reports in the literature, it is suggested that meta-analysis may better establish this possible association.

In the paper entitled “*What is really risky in melanoma? prognostic parameters for the primary care of melanoma patients*,” D. Göppner and M. Leverkus reviewed the literature data and summarized current understanding of carcinogenesis in melanoma giving a detailed overview of known morphologic and potentially future genetic prognostic parameters in malignant melanoma.

The paper entitled “*Treatment of locally advanced melanoma by isolated limb infusion with cytotoxic drugs*” overviews isolated limb infusion (ILI), as a less invasive technique than the classical isolated limb perfusion (ILP) which may be preferred for locally advanced melanoma confined to a limb. The minimally invasive character of ILI may replace ILP in the future as palliation for locally advanced limb tumors.

In the paper entitled “*Characterization of ex vivo expanded tumor infiltrating lymphocytes from patients with malignant melanoma for clinical application*,” M. H. Andersen et al. report a different method for expanding tumor infiltrating lymphocytes (TIL) to clinically relevant quantities in two steps within 8 weeks. This method may be utilized for new clinical trials, where adoptive transfer of autologous tumor infiltrating lymphocytes (TIL) has shown objective clinical responses in up to 50% of treated patients. 

In the paper entitled “*Management of melanoma brain metastases in the era of targeted therapy*,” D. G. Shapiro and W. E. Samlowski discuss approaches for melanoma brain metastases using targeted agents combined with classical surgery and radiosurgery, along with the possibility of improving survival in at least a subset of melanoma patients with brain metastases.

In the paper entitled “*Uveal melanoma*,” V. M. L. Cohen and V. P. Papastefanou give a 360°C overview uveal melanoma from genetic alterations to diagnosis and treatment. 

These studies span the range of etiology and regional as well as systemic therapy for melanoma, to provide a background that may be useful for readers in relation to regional and systemic metastasis, including the most ominous phase of brain metastasis, which punctuate the course of disease. The clear differences between uveal melanoma, a disease nominally included among ‘‘the melanomas” are now biologically recognized as diverse in histogenesis and relevant mutational oncogenic pathways. These papers are timely in this year of regulatory approvals for several new agents, and the pending likelihood that new agents will join this armamentarium for melanoma. An area not touched upon is that of adjuvant therapy, which may provide insights to further roles of therapy and ultimately, with chemoprevention, may lessen the burden of advanced disease in the future. 

*Paolo A. Ascierto* *Paolo A. Ascierto* *John M. Kirkwood* *John M. Kirkwood* *Francesco M. Marincola* *Francesco M. Marincola* *Giuseppe Palmieri* *Giuseppe Palmieri*

